# Construction of an SSR and RAD-Marker Based Molecular Linkage Map of *Vigna vexillata* (L.) A. Rich

**DOI:** 10.1371/journal.pone.0138942

**Published:** 2015-09-23

**Authors:** Rusama Marubodee, Eri Ogiso-Tanaka, Takehisa Isemura, Sompong Chankaew, Akito Kaga, Ken Naito, Hiroshi Ehara, Norihiko Tomooka

**Affiliations:** 1 Graduate School of Bioresources, Mie University, 1577 Kurimamachiya-cho, Tsu, Mie, 514–0102, Japan; 2 Department of Plant Science and Agricultural Resources, Faculty of Agriculture, Khon Kaen University, Khon Kaen, 40002, Thailand; 3 Genetic Resources Center, National Institute of Agrobiological Sciences, Kannondai 2-1-2, Tsukuba, Ibaraki, 305–8602, Japan; National Key Laboratory of Crop Genetic Improvement, CHINA

## Abstract

*Vigna vexillata* (L.) A. Rich. (tuber cowpea) is an underutilized crop for consuming its tuber and mature seeds. Wild form of *V*. *vexillata* is a pan-tropical perennial herbaceous plant which has been used by local people as a food. Wild *V*. *vexillata* has also been considered as useful gene(s) source for *V*. *unguiculata* (cowpea), since it was reported to have various resistance gene(s) for insects and diseases of cowpea. To exploit the potential of *V*. *vexillata*, an SSR-based linkage map of *V*. *vexillata* was developed. A total of 874 SSR markers successfully amplified single DNA fragment in *V*. *vexillata* among 1,336 SSR markers developed from *Vigna angularis* (azuki bean), *V*. *unguiculata* and *Phaseolus vulgaris* (common bean). An F_2_ population of 300 plants derived from a cross between salt resistant (V1) and susceptible (V5) accessions was used for mapping. A genetic linkage map was constructed using 82 polymorphic SSR markers loci, which could be assigned to 11 linkage groups spanning 511.5 cM in length with a mean distance of 7.2 cM between adjacent markers. To develop higher density molecular linkage map and to confirm SSR markers position in a linkage map, RAD markers were developed and a combined SSR and RAD markers linkage map of *V*. *vexillata* was constructed. A total of 559 (84 SSR and 475 RAD) markers loci could be assigned to 11 linkage groups spanning 973.9 cM in length with a mean distance of 1.8 cM between adjacent markers. Linkage and genetic position of all SSR markers in an SSR linkage map were confirmed. When an SSR genetic linkage map of *V*. *vexillata* was compared with those of *V*. *radiata* and *V*. *unguiculata*, it was suggested that the structure of *V*. *vexillata* chromosome was considerably differentiated. This map is the first SSR and RAD marker-based *V*. *vexillata* linkage map which can be used for the mapping of useful traits.

## Introduction

*Vigna vexillata* (L.) A. Rich. is a perennial herb belonging to the genus *Vigna* and considered to be closely related to cowpea (*Vigna unguiculata* (L.) Walp.), the most important food legume in Africa [1]. A domesticated form of *V*. *vexillata*, which has large non-dormant seeds and non-shattering pods named “tuber cowpea”, was found cultivated for their flesh tubers and mature seeds in Bali and Timor, Indonesia [2]. Another cultivated form *V*. *vexillata* var. *macrosperma* was described from the specimen from Costa Rica [3]. The protein content of tuberous roots of *V*. *vexillata* is about three times higher (15%) than that of potato and about six times higher than that of cassava [4]. The tuberous roots of wild *V*. *vexillata* have been eaten like sweet potato by local people in Ethiopia and Sudan [5], in Himalayas and in the hills of eastern and northeastern India at altitude between 1,200 and 1,500m [6,7] and in tropical and subtropical Australia [8]. The wild plant has been used also as forage or cover crop in several African countries [9,10] and in Australia [11].

The wild *V*. *vexillata* distributed from Africa, Asia, Australia to Central and South America. Due to its worldwide distribution, *V*. *vexillata* shows a great morphological variation and several botanical varieties are recognized [3,12–14]. Wild *V*. *vexillata* is considered to be an important gene(s) source in cowpea breeding [15], because some of its accessions are highly resistant to the cowpea weevil [16], pod sucking bugs, flower thrips, *Maruca vitrata* (Fabricius) [17] and *Striga gesnerioides* (Scrophulariaceae) [18], powdery mildew [19] and cowpea mottle carmovirus [1,20,21]. In addition, it shows high levels of resistances to various environmental stresses, such as to prolonged water logging [11], to lateritic acid and aluminous soils [6], to infertile sandy loams to fertile heavy-textured clays and to alkaline cracking clay soil [8,22].

Genetic mapping is a powerful approach to understand the function of genes in a variety of biological processes [23]. Discovering genes that control morphological and physiological phenotypes is critical for understanding the mechanism of adaptive evolution and for plant breeding [24]. The SSR (simple sequence repeat) markers have particular advantages for characterization and mapping of gene(s) because of their high reproducibility, co-dominant inheritance, relative abundance, high polymorphism, and ease of genotyping [25]. The SNP (single nucleotide polymorphisms) markers have been developed based on genome sequence information and have been used for mapping gene(s). Among several methods of developing SNP markers, SNP discovery using sequenced RAD (Restriction site Associated DNA) markers have advantages for non-model organisms without prior reference genome sequence information [26,27]. By using the high-throughput sequencing of DNA fragments flanking the restriction sites (RAD tags), a huge number of SNPs could be identified as co-dominant markers efficiently.

To date, several SSR-based linkage maps of *Vigna* crops, including azuki bean (*V*. *angularis* (Willd.) Ohwi & H. Ohashi: [28], [29], [30]), black gram (*V*. *mungo* (L.) Hepper: [31]), mungbean (*V*. *radiata* (L.) R. Wilczek: [32]), rice bean (*V*. *umbellata* (Thunb.) Ohwi & H. Ohashi: [33]) and yardlong bean (*V*. *unguiculata*: [34]) were reported. Based on the comparative genome mapping study using the common SSR markers, it was revealed that all the four *Vigna* crops belonging to the subgenus *Ceratotropis*, i. e., azuki bean, black gram, mungbean and rice bean have highly conserved genome structures, and therefore the QTLs of domestication related traits among these 4 *Ceratotropis* crops could successfully be compared [35].

Recently, two sequencing-based linkage maps of azuki bean and mungbean [36,37]) were reported. However, there are no reports of the linkage map of *V*. *vexillata* consist of the 11 linkage groups which correspond to the number of chromosomes of this promising crops for the future [1,35].

Hence, the objectives of this study were to construct linkage map of *V*. *vexillata* (1) using SSR markers developed from related *Vigna* and *Phaseolus* crops, (2) using RAD markers developed by *de novo* RAD-sequencing and (3) to compare constructed linkage map with those of two most important *Vigna* crops, *V*. *radiata* and *V*. *unguiculata*.

## Materials and Methods

### Plant materials

An F_2_ mapping population was developed from a cross between wild *V*. *vexillata* accession V1 (from USDA, PI 406383, duplicate conserved as JP202334 in the NIAS genebank, National Institute of Agrobiological Sciences, Japan) and V5 (from the Botanic Garden, Meise, NI 936, conserved as JP235869). V1 is a salt tolerant accession collected from Paramaribo, Suriname while V5 is a salt sensitive accession collected at a site 35km E of Santa Marta, Columbia. The V1 was used as a male parent and V5 was a female parent in the cross to produced F_1_ seeds. The F_1_ plant was self-pollinated to produce 300 F_2_ plants which were grown in a greenhouse of NIAS from May 2013.

### DNA extraction

Total genomic DNA of the parents and F_2_ plants were extracted from fresh leaf tissue using the CTAB method [38] with a slight modification. The DNA was adjusted to 5 ng/μl for SSR marker analysis by comparing with known concentrations of standard λ–DNA on 1.5% agarose gel.

### SSR marker analysis

A total of 1336 SSR markers consisting of 329 SSR markers of azuki bean [39], 480 EST-SSR markers of azuki bean [40], 487 SSR markers of cowpea [34,41], and 40 SSR markers of common bean [42–45] were screened (Table 1, S1 Table). For azuki bean SSR, common bean SSR, cowpea SSR (VM primers; Li et al. [41]), each PCR reaction mixture solution was prepared to a volume of 5 μL containing 5 ng DNA, 1x QIAGEN Multiplex PCR Master Mix and 5 pmol of forward and reverse primers. The 5’-end of the reverse primer was fluorescent labeled with one of the four following fluorescent dyes: 6-FAM (blue), VIC (green), NED (yellow) and PET (red) (Applied Biosystems). For azuki bean EST and cowpea SSR (cp primers; Kongjaimun et al. [34]), each PCR reaction mixture solution was prepared to a volume of 5 μL containing 5 ng DNA, 1x QIAGEN Multiplex PCR Master Mix, 1x Q-solution, 2 pmol of forward primer and 20 pmol of reverse primer. The 5’-end of the forward primer was fluorescent labeled with one of the three following fluorescent dyes: FAM (blue), HEX (green) and NED (yellow) (Applied Biosystems). PCR reactions were performed in a GeneAmp PCR System 9700 (Applied Biosystems) The PCR thermal cycling was programmed as follows: 95°C for 15 mins follow by 40 cycles of 94°C for 30 s, 55°C for 90 s, 72°C for 60 s, and a final cycle at 72°C for 10 mins. For azuki bean SSR, common bean SSR, cowpea SSR (VM primers; Li et al. [41]), 1 μL of ten times diluted PCR product was mixed with 8.5 μL of Hi-Di formamide and 0.125 μL of Gene Scan 500 LIZ size standard (Applied Biosystems). For azuki bean EST and cowpea SSR (cp primers; Kongjaimun et al. [34]), 1 μL of five times diluted PCR product was mixed with 8.5 μL of Hi-Di formamide and 0.125 μL of Gene Scan 500 ROX size standard (Applied Biosystems). The mixer was denatured at 95°C for 5 mins and run on an ABI Prism 3100 or 3130xl Genetic Analyzer (Applied Biosystems). Allele size for the highest stutter peak with the height ranging between 500 and 10,000 RFU was recorded and used to create bins for automatic assignment of genotypes. The genotyping was conducted by the GeneMapper 3.0 software (Applied Biosystems) with default settings. After marker screening, 6 or 7 primers with different labels and product sizes were put into a single PCR reaction mixture and amplified as a multiplex PCR using the same procedures described above.

**Table 1:** Summary of amplification of SSR and EST-SSR markers from three legumes in *Vigna vexillata* and polymorphic rate between parents V1 and V5.

SSR sources	Marker type	Screened	Amplified (%)^b^	Polymorphic (%)^c^
Azuki bean	SSR^a^	329	257 (78.1)	28 (8.5)
Azuki bean	EST-SSR	480	305 (63.5)	19 (4.0)
Cowpea	SSR	487	273 (56.1)	35 (7.2)
Common bean	SSR	40	39 (97.5)	1 (2.5)
Total		1,336	874 (65.4)	83 (6.2)

^a^ One STS marker is included.

^b^ (No. of SSR and EST-SSR primer pairs amplified/ No. of SSR and EST-SSR primer pairs screened)*100

^c^ (No. of SSR and EST-SSR primer pairs polymorphic/ No. of SSR and EST-SSR primer pairs screened)*100.

### SSR linkage map construction and comparison with related species

An SSR genetic linkage map was constructed with JoinMap ver. 4.0 [46]. The calculation was set with a minimum logarithm of the odds (LOD) of 4.0 and a maximum recombination frequency (*r*) of 0.25. Kosambi mapping function [47] was used to calculate the distance between SSR loci. For each marker, chi-square analysis was calculated for goodness of fit to a 1:1 segregation ratio of genotypic classes at P = 0.05, 0.01, and 0.001. Markers were assigned to an LG based on recombination frequencies and LOD values. The recombination frequencies were converted into map distances (cM) using the mapping function of Kosambi [47]. Double crossovers between adjacent loci were confirmed manually. The numbering of linkage groups was named following mungbean linkage map [32]. Based on common SSR markers, the structure of the linkage maps among mungbean (*V*. *radiata*), yardlong bean (*V*. *unguiculata*) and *V*. *vexillata* were compared. In case more than 2 SSR markers of a mungbean linkage group (e.g., LG1) were mapped with several SSR markers of another mungbean linkage group (e.g., LG5), it is estimated that a translocation is occurred.

### RAD-seq analysis

RAD-seq analysis was performed based on the protocol of Matsumura et al. with minor modifications [48].

### Preparation of adaptors

Adapter-1 for BamHI-digested DNA site was prepared by annealing the two synthesized oligonucleotides 5’-biotin-GTACAGGTTCAGAGTTCTACAGTCCGACGATCXXXXXX-3′ and 5′-GATCXXXXXXGATCGTCGGACTGTAGAACTCTGAACCTGT-3 (XXXXXX correspond to the variable index sequences to identify the individual DNA sample). Adapter 2 for NlaIII-digested DNA site was prepared by annealing of the two complementary oligonucleotides 5′-amino-CAAGCAGAAGACGGCATACGACATG-3′ and 5′-TCGTATGCCGTCTTCTGCTTG-3′.

### Library construction and RAD sequencing

DNA library for RAD sequencing was constructed as follows. Each genomic DNA samples (100–300 ng) of parents and 286 F_2_ individuals were simultaneously digested with BamHI-HF and NlaIII and purified. Adaptor-1 and adaptor-2 were ligated to the digested DNA samples and purified. Adaptors ligated biotinylated DNA samples were collected using streptavidin coated magnetic beads (Dynabeads M270, Dynal). Adaptor ligated DNA on the beads was amplified by PCR using Phusion High-Fidelity DNA polymerase (Thermo Fisher Scientific) and the adapter primers. Size of the PCR amplified fragments were checked by electrophoresis in agarose gel. The 96 purified PCR products were pooled and sequenced using the Illumina HiSeq2000 system. The sequencing primer was 5′-CGACAGGTTCAGAGTTCTACAGTCCGACGATC.

### Extraction of RAD-tag and bi-allelic RAD-marker detection

Extraction of RAD-tag sequence and bi-allelic RAD-marker detection were conducted using a software, Stacks ver. 1.12 [49]. Sequence reads of low quality and sequence reads with ambiguous six-base variable index sequences were discarded and 85 bp RAD-tag sequence reads were prepared. The RAD-tag sequence reads were classified into those of 2 parental accessions and F_2_ individuals based on the six-base variable index sequences. RAD-tags with the sequence reads of less than 2 sequence mismatch were grouped as a stack. Stacks of each parent with less than 3 sequence mismatch were estimated as the stacks derived from a homologous locus. A list of RAD-tag sequences and their count was constructed for each sample. For the genotyping of F_2_ individuals, stacks which have more than 20 RAD-tags (minimum stack depth of 20) were used as potential RAD-markers.

### Linkage map construction using SSR and RAD-markers

Linkage group construction was conducted by a software R/qtl ver. 1.36.6 [50]. Potential RAD-markers which have genotype data of less than 100 F_2_ individuals, which show identical F_2_ individuals genotypes, and/or which show significant segregation distortion (chi-squared test, P < 10^−5^) were not used. Suitable values of recombination fractions and LOD scores were estimated using the est.rf command. After these steps, initial linkage group construction was carried out using the formLinkageGroups command with a maximum recombination fraction of 0.25 and LOD threshold of 15. Robustness of linkage groups was checked using plot.rf command. Marker order within a linkage group was estimated using a program TMAP [51]. Some RAD-markers mapped at less than 0.1cM locus were not used for linkage map construction.

## Results

### Linkage map based on SSR markers

Among 1,336 SSR markers screened, 874 SSR markers (65.4% on average) amplified single DNA fragment (Table 1). The percentage of SSR markers amplified ranged from 56.1% for cowpea SSR markers to 97.5% for common bean SSR markers. Among 874 amplified SSR markers, only 83 SSR markers (6.2%) showed polymorphism between parents. The percentage of polymorphic SSR markers ranged from 2.5% for common bean SSR markers to 8.5% for azuki bean SSR markers.

**Fig 1:**
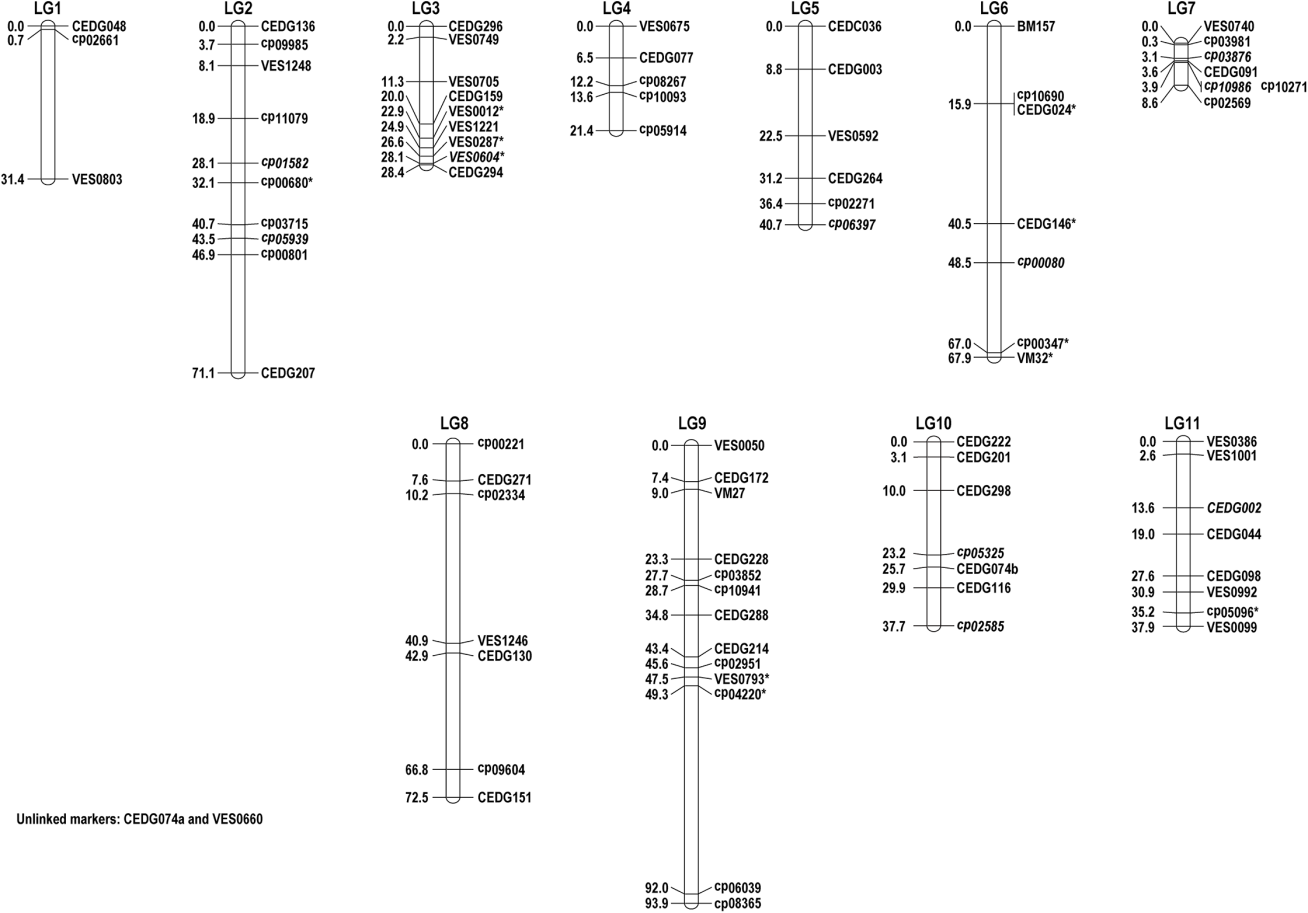
A genetic linkage map of *V*. *vexillata* constructed from 300 F_2_ individuals of intraspecific cross between accessions V1 and V5. Map distances and marker names are shown on the left and right side of the linkage groups, respectively. Marker names in italics indicate dominant loci. Markers showing significant deviation from the expected segregation ratio at 0.05 are indicated with*. SSR markers with prefixes CED are derived from azuki bean. Markers with prefixes cp and VM are derived from cowpea. BM157 (LG6) is derived from common bean. Markers with prefixes VES are EST-SSR markers derived from azuki bean.

A total of 82 out of 84 polymorphic SSR loci could be assigned to 11 linkage groups (LGs) covering a total length of 510.5 cM of the *V*. *vexillata* genome at an average marker distance of 7.2 cM (Fig 1, Table 1). Two polymorphic markers (CEDG074a and VES0660) were unlinked and could not be mapped. The number of markers on each LG ranged from 3 (LG1) to 13 (LG9) (Table 2). The length of each LG ranged from 8.6 (LG7) to 93.9 cM (LG9). The average distance between two adjacent markers ranged from 1.4 (LG7) to 15.7 cM (LG1). The LGs 1, 2, 6, 8 and 9 had gaps greater than 15 cM between markers. Eight markers (9.8%) showed significant segregation distortion at 5% level (Fig 1).

**Table 2:** Comparison of *Vigna vexillata* linkage group with those of *V*. *radiata* and *V*. *unguculata*, and some characteristics of the constructed *V*. *vexillata* SSR linkage map.

Linkage	Corresponding linkage groups of	Length			No. of loci			Average distance between two maker loci
group	*V*. *radiata* and *V*. *unguiculata* maps	(cM)	Azuki bean SSR	Cowpea SSR	Common bean SSR	Azuki bean EST-SSR	Total	(cM)
1	1	31.4	1	1	0	1	3	15.7
2	2	71.1	2	7	0	1	10	7.9
3	3	28.4	3	0	0	6	9	3.6
4	4	21.4	1	3	0	1	5	5.4
5	1 and 5	40.7	3	2	0	1	6	8.1
6	6 and 9	67.9	2	4	1	0	7	11.3
7	4	8.6	1	5	0	1	7	1.4
8	8	72.5	3	3	0	1	7	12.1
9	1 and 9	93.9	4	7	0	2	13	7.8
10	10	37.7	5	2	0	0	7	6.3
11	11	37.9	3	1	0	4	8	5.4
**Total**	**-**	**511.5**	**28**	**35**	**1**	**18**	**82**	**7.2**

Unlinked markers: CEDG074a and VES0660

### Linkage map based on SSR and RAD markers

Illumina sequencing with HiSeq2000 yielded a total of 451,012,199 RAD-tag 85-base reads from 494,666,849 raw reads. The number of RAD-tags of parents (V1 and V5) and F_2_ individuals was 412,372, 1,138,645 and 450,040,150, respectively (Table 3). The average number of RAD-tags per F_2_ individual was 1,597,514.3. RAD-tags were aligned and clustered into 42,517 stacks. Among these stacks, 10,037 candidate RAD loci were inferred and genotyped for 286 individuals of F_2_ population. For the analysis of the F_2_ mapping population, 5,438 RAD markers which showed homozygote polymorphic genotype between parents were used. Among them, 735 RAD markers together with 84 SSR markers loci were used for the linkage map construction after discarding markers showing identical F_2_ genotypes, high level of segregation distortion, and less than 100 F_2_ genotypes.

**Table 3:** RAD-seq results in parents and F_2_ populations using HiSeq2000 platform.

Samples	Number of reads	Number of RAD-tag	Mean coverage depth	Mean merged coverage depth
*V*. *vexillata* V1	442,165	412,372	12.0	12.7
V5	1,176,089	1,138,645	24.0	25.2
F_2_ populations (286 lines)	470,584,049	450,040,150	-	-
Average per F_2_ individual	1,670,491	1,597,514.3	30.6	37.0

**Fig 2:**
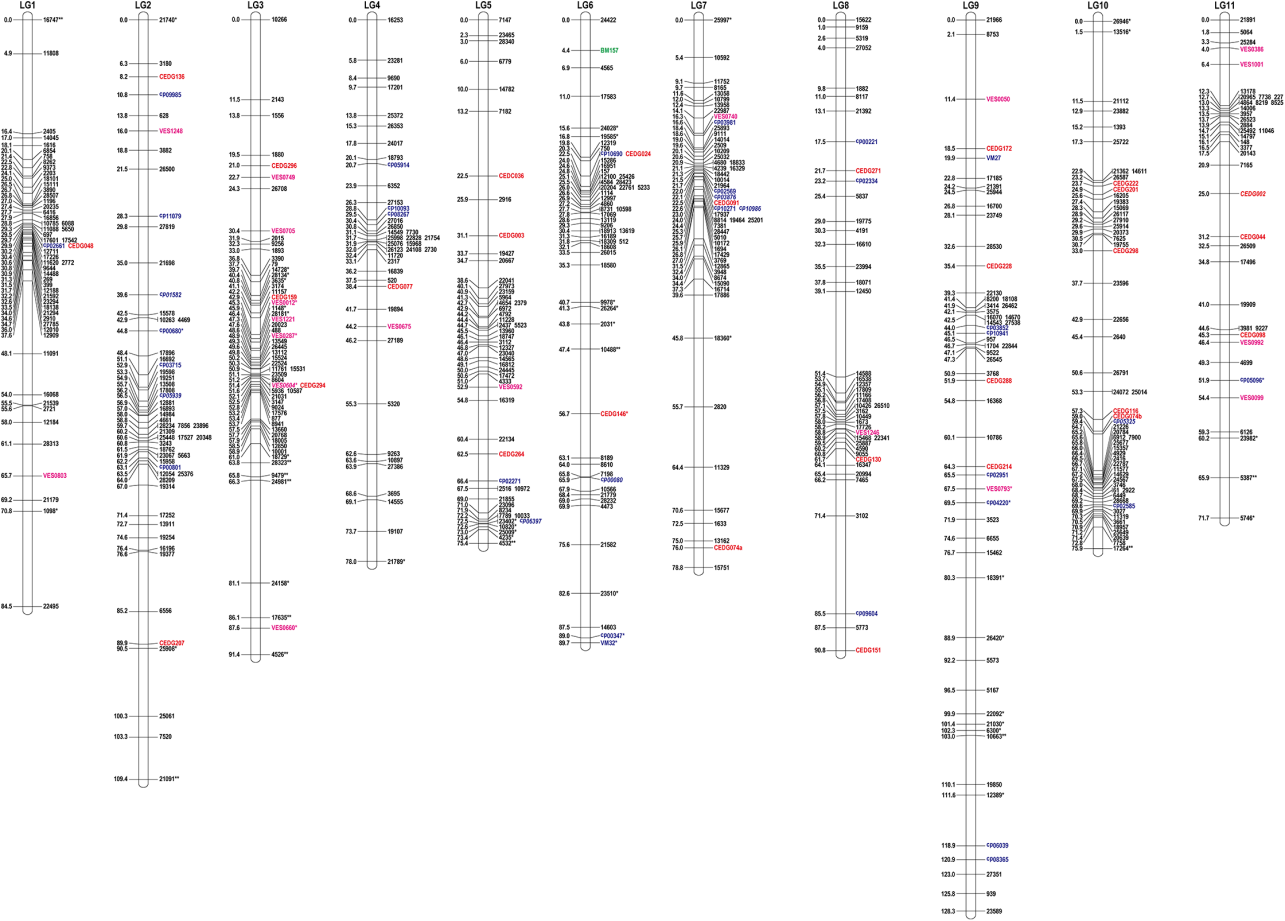
The SSR and RAD markers linkage map of *V*. *vexillata* constructed from 286 F_2_ individuals of intraspecific cross between accessions V1 and V5. Map distances and marker names are shown on the left and right side of the linkage groups, respectively. Marker names in italics indicate dominant loci. Markers showing significant deviation from the expected segregation ratio at 0.05 and at 0.01 are indicated with* and **, respectively. SSR markers with prefixes CED are derived from azuki bean (red text). Markers with prefixes cp and VM are derived from cowpea (blue text). BM157 (LG6) is derived from common bean (green text). Markers with prefixes VES are EST-SSR markers derived from azuki bean (purple text). Markers without prefixes are RAD-markers derived from *V*. *vexillata* (black text).

Linkage analysis identified 11 linkage groups (LG1–LG11) containing a total of 475 RAD markers loci and 84 SSR markers loci after the removal of unlinked markers (Table 4, Fig 2). Sequence and SNP position of each RAD-marker was summarized (S2 Table). The map spanned 973.9 cM, with a mean distance between markers of 1.8 cM. The lengths of linkage groups ranged from 71.7 (LG11) to 128.3 cM (LG9). The positions of all SSR markers in an SSR-RAD markers linkage map were consistent with those in SSR markers linkage map.

**Table 4:** Characteristics of a *V*. *vexillata* linkage map based on the SSR and RAD markers.

Linkage group		Number of loci		Length (cM)	Average marker interval (cM)
	Total	SSR	RAD		
1	54	3	51	84.5	1.6
2	56	10	46	109.4	2.0
3	58	10	48	91.4	1.6
4	41	5	36	78.0	1.9
5	50	6	44	75.4	1.5
6	54	7	47	89.7	1.7
7	53	8	45	78.8	1.5
8	43	7	36	90.8	2.2
9	55	13	42	128.3	2.4
10	56	7	49	75.9	1.4
11	39	8	31	71.7	1.9
Total	559	84	475	973.9	1.8

### Comparative linkage maps

**Fig 3:**
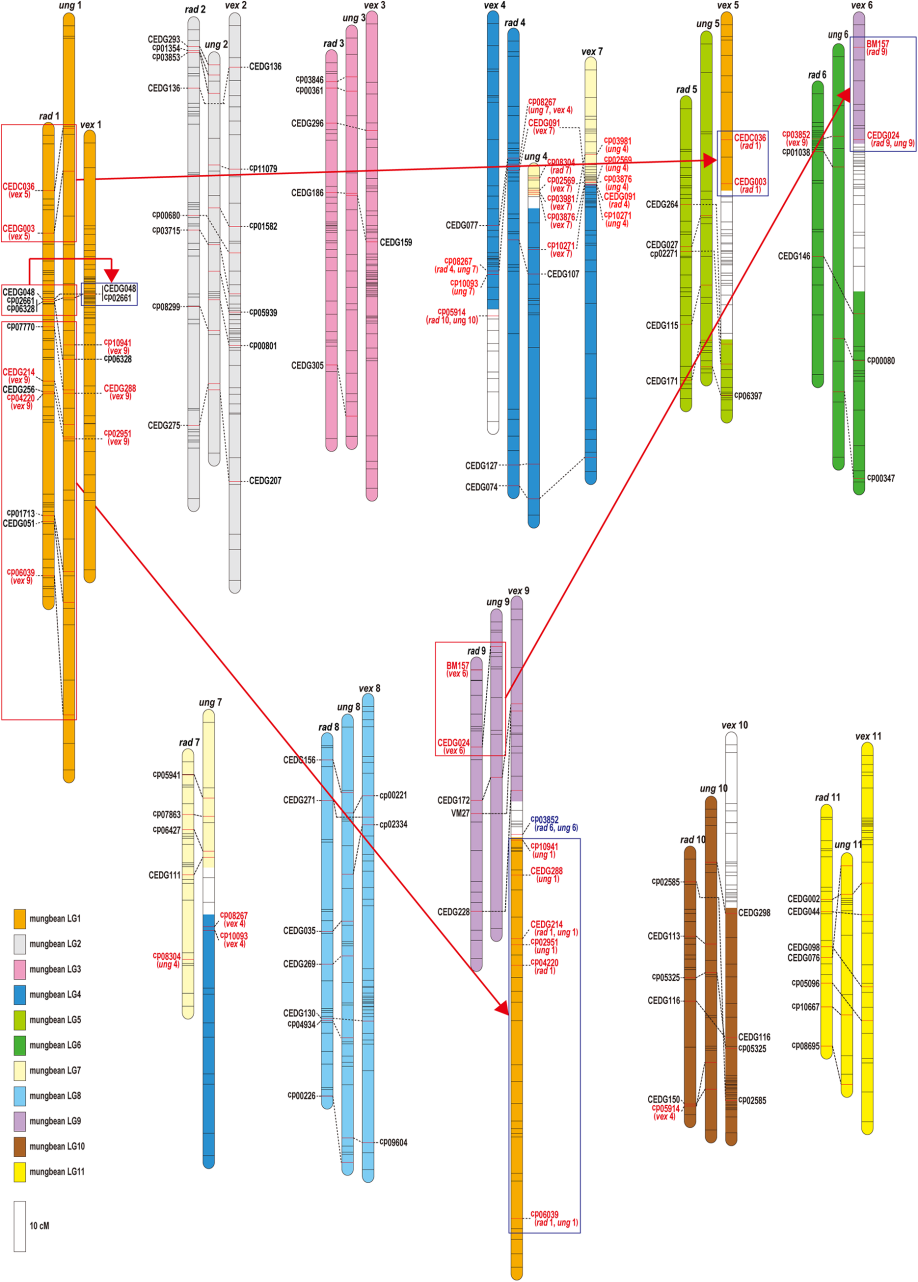
Comparative linkage maps among *Vigna radiata* (left: rad), *V*. *unguiculata* (middle: ung), and *V*. *vexillata* (right: vex) based on common SSR markers. Linkage group 4 (LG 4) of *V*. *vexillata* (vex 4) was placed to the left and LG 7 of *V*. *vexillata* (vex 7) was placed to the right of LG4 of *V*. *radiata* (rad 4) to show the relationships of common markers. The positions of common markers were connected by dotted lines. Markers which were mapped on different LGs among 3 *Vigna* species were written in red text. Major translocations were indicated by red arrows. The species abbreviation name and number in parentheses below red text marker names, e.g., (vex 5) indicate that the species and linkage group number on which corresponding marker was mapped.

Comparative linkage maps based on the common SSR markers were developed (Fig 3). Linkage maps of *V*. *radiata* (left) and *V*. *unguiculata* (middle) were highly conserved. Most of the common markers were mapped on the same linkage group with the same order. One reciprocal translocation was found between LG4 and LG7. On the other hand, 3 translocations were detected in *V*. *vexillata* compared with *V*. *radiata* (Fig 3, Table 2). Among 11 LGs, the structure of 8 *V*. *vexillata* LGs (vex LGs 1, 2, 3, 4, 7, 8, 10, 11) was estimated to be conserved with those of *V*. *radiata* and *V*. *unguiculata*. Other 3 LGs of *V*. *vexillata* (vex LGs 5, 6, 9) were composed of the fragments of 2 LGs of *V*. *radiata* or *V*. *unguiculata*.

## Discussion

### Transferability of SSR markers

The genetic linkage map developed in this study is the first SSR and RAD markers-based linkage map of *V*. *vexillata*. In addition, this is the first genetic linkage map consisting of 11 linkage groups that correspond to the haploid chromosome number of *V*. *vexillata* (n = 11). Former *V*. *vexillata* linkage map was constructed by 70 RAPD, 47 AFLP and one SSR marker, and was consisted of 14 linkage groups [1].

SSR markers developed in related species of *Vigna* and *Phaseolus* showed good amplification and a total of 874 markers were selected as potentially useful markers for *V*. *vexillata* (Table 1, S1 Table). However, there were only 83 polymorphic markers between the parents (V1 and V5). This might be explained by the fact that both of the parental accessions were from South America (V1 from Suriname and V5 from Columbia), where the genetic diversity of this species was reported to be low [3]. Baudoin and Maréchal [52] mentioned that there were two main centers of diversity, one in eastern and southern Africa (Zambezian district) and the other in Southeast Asia from Yunnan to Indonesia. Spinosa et al. [53] showed a lower degree of isozyme and RAPD variations in American accessions compared with African accessions. Vanderborght [10] reported that accessions from America showed epigeal germination which might be a recent evolutionary trend since most of the African accessions showed hypogeal germination which is considered ancestral type of germination in *V*. *vexillata*.

### RAD-seq analysis for linkage map construction

SSR markers have been used as useful molecular markers and several SSR based linkage maps were constructed for *Vigna* crops [28–34]. However, we have encountered a problem of low polymorphism in SSR markers (6.2%) between *V*. *vexillata* parental accessions, hence resulting in a low density SSR linkage map. Recently, RAD-markers have been successfully used to construct a genetic linkage map and to perform QTL mapping in higher plant species [54–56].

By the application of RAD-seq analysis to the genetically close parental accessions of *V*. *vexillata* (V1 and V5), we could construct a high-density linkage map with 11 linkage groups with 475 RAD-markers (SNPs based of RAD-tag sequence) (S2 Table). Recently, a mungbean (*V*. *radiata*) and an azuki bean (*V*. *angularis*) linkage maps were constructed by SNPs using GBS method (genotyping-by-sequencing) [35,36]. However, since they used different enzyme (ApeKI) in library preparation, sequence of the DNA fragments flanking the restriction sites could not be compared with RAD-tag sequence of the present study.

Since the genetic linkage map based on the RAD and SSR markers could confirm the linkage group accuracy of SSR-based linkage map, comparative genomic analysis were performed using common SSR markers.

### Translocations in *Vigna vexillata*

The present study suggested several translocations occurred in *V*. *vexillata* chromosomes, while genomic structure of *V*. *radiata* and *V*. *unguiculata* was basically conserved with only one translocation. The remarkable chromosomal re-arrangements in *V*. *vexillata* was surprising because molecular phylogenetic analysis based on RFLP and the sequences of nuclear ribosomal DNA (rDNA) regions suggested that *V*. *vexillata* was more closely aligned with *V*. *unguiculata* compared with *V*. *radiata* [18,57,58]. In addition, morphological studies suggested *V*. *vexillata* group (subgenus *Plectrotropis*) formed an evolutionary intermediate group between *V*. *unguiculata* group (subgenus *Vigna*) and *V*. *radiata* group (subgenus *Ceratotrois)* [52,59].

Similar phenomenon was reported in the comparative analyses among *V*. *marina*, *V*. *radiata* and *V*. *unguiculata* linkage maps [60]. Structure of linkage maps was completely conserved between *V*. *marina* and *V*. *radiata*, which belong to the different subgenera, i.e., subgenus *Vigna* and *Ceratotropis*, respectively. On the contrary, one reciprocal translocation between LG4 and 7 was found between *V*. *marina* and *V*. *unguiculata*, which belong to the same subgenus *Vigna*. However, since the number of common SSR markers used for developing the comparative linkage map was not sufficient in these studies, it should be confirmed by the comparison of higher density molecular maps or more directly by cytogenetic analysis. Gomathinayagam et al. [61] observed the meiotic chromosomes of a hybrid of *V*. *vexillata* x *V*. *unguiculata* and reported high frequency of univalent formation, hence suggested that the genomes of the two species are structurally differentiated.

In addition, it is unknown that the chromosomal re-arrangement of *V*. *vexillata* suggested in the present study is commonly seen in *V*. *vexillata* or only occurred in some local accessions (e.g. American accessions). In case of azuki bean (*V*. *angularis*), we also suggested reciprocal translocation between LG4 and LG6 based on the comparison of SSR-based linkage maps developed for 2 different mapping populations [29,30]. Recently, this translocation event was confirmed to be occurred on a Japanese wild azuki bean parent by BAC-fluorescence in situ hybridization (FISH) analyses [62]. In that study, 21 wild azuki bean accessions collected from various regions of Japan were analyzed and it was found that geographical distribution of accessions with translocated chromosomes were restricted to eastern and northern Japan.

### Future perspectives

*Vigna vexillata* is a promising food crop which could be grown under harsh environmental conditions [63]. There are domesticated accessions collected in Bali, which could be harvested in about 3 months and estimated tuber yield is 18–30 t/ha and seed yields of 0.7–1.2 t/ha [2]. There is another domesticated form (var. *macrosperma*) recognized having large seeds, non-dehiscent pods and bushy plant type [3,64]. *V*. *vexillata* var. *macroperma* showed earlier maturity with higher seed yield than Bali cultivated accessions [64]. Based on the hybridization study of these 2 domesticated forms (Bali and var. *macrosperma*) with African and Austronesian wild *V*. *vexillata* accesions, it was revealed that var. *macrosperma* and the wild accessions can be considered to belong to the same gene pool with strong genetic compatibility [65]. In contrast, there were various levels of genetic barriers between the cultivated Bali accessions and the wild accessions. Among several cross combinations tried, there was an Australian wild accession which could produce F_1_ plants with a cultivated Bali accession having vigorous growth, but it showed complete F_1_ sterility. However, backcrossing both to wild and Bali cultivated parents could produce BC_1_ plants, suggesting the possibility of backcross breeding of improving these domesticated forms [66]. As was mentioned in Introduction section, there are wild accessions showing high levels of resistance to various kinds of insects and diseases, and also to various kinds of abiotic stresses. Therefore, diverse wild *V*. *vexillata* could be used to incorporate adaptation gene(s) into these domesticated forms to grow under both biotic and abiotic stress environments.

In the breeding procedure, especially using wild germplasm, it is essential to map the position of useful traits and use marker assisted selection in the segregating population [67]. The SSR and RAD-marker based linkage map and SSR markers which produce single amplified DNA fragment in *V*. *vexillata* in the present study could be used as basic genome mapping resources.

## Supporting Information

S1 Table SSR and EST-SSR primers information. (XLSX)

S2 Table RAD markers name and sequence. (XLSX)
